# Changes in alanine aminotransferase in adults with severe and complicated obesity during a milk-based meal replacement programme

**DOI:** 10.1186/s12986-020-00512-5

**Published:** 2020-10-16

**Authors:** Razk Abdalgwad, Mohammed Faraz Rafey, Conor Murphy, Iulia Ioana, Paula Mary O’Shea, Eoin Slattery, Colin Davenport, Derek Timothy O’Keeffe, Francis Martin Finucane

**Affiliations:** 1 Bariatric Medicine Service, Centre for Diabetes, Endocrinology and and HRB Clinical Research Facility, Galway University Hospitals, Galway, Ireland; 2 HRB Clinical Research Facility, National University of Ireland Galway, Galway, Ireland; 3 Department of Medicine, National University of Ireland Galway, Galway, Ireland; 4 Department of Clinical Biochemistry, Galway University Hospitals, Galway, Ireland

**Keywords:** Non-alcoholic fatty liver disease, Non-alcoholic steatohepatitis, Alanine aminotransferase, Severe obesity, Milk-based meal replacement programme, Bariatric

## Abstract

**Introduction:**

Excess adiposity is associated with fat accumulation within the liver, and non-alcoholic steatohepatitis (NASH) is highly prevalent in bariatric patients. Elevated alanine aminotransferase (ALT) is associated with prevalent NASH. We sought to determine the influence of a milk-based meal replacement weight-loss programme on ALT levels in adults with severe and complicated obesity.

**Methods:**

We conducted a retrospective cohort study of patients who completed a 24-week meal replacement programme, comprised of a weight loss phase followed by weight stabilisation and maintenance phases, each 8 weeks long. ALT was quantified using an enzymatic assay with spectrophotometric detection. We examined changes over time in ALT using the non-parametric Wilcoxon singed-rank test and the Friedman test.

**Results:**

Of 105 patients, 56 were female, mean age was 51.2 ± 11.2 (range 18.0–71.6) years. There was an unanticipated but transient increase in ALT from 28.0 [20.0, 40.5] iu/L at baseline to 40.0 [26.0, 55.0] iu/L after 2 weeks (*p* < 0.0005), followed by a gradual reduction to 21.0 [17.0, 28.3] iu/L by 24 weeks (*p* < 0.0005). The overall reductions in ALT were more pronounced in patients who had elevated levels at baseline. Body weight decreased from 144.2 ± 28.0 kg at baseline to 121.6 ± 25.4 kg at 24 weeks (*p* < 0.0005) and body mass index (BMI) decreased from 50.7 ± 8.1 kg m^−2^ at baseline to 43.0 ± 7.6 kg m^−2^ by 24 weeks (*p* < 0.0005).

**Conclusion:**

In adults with severe and complicated obesity undergoing a milk-based meal replacement programme, there was an initial unanticipated rise in ALT in the first 2 weeks, followed by a gradual overall reduction by 24 weeks. These findings suggest that rapid weight loss secondary to significant caloric restriction might induce a transient deterioration in hepatic steatosis prior to an ultimate overall improvement.

## Introduction

Alanine aminotransferase (ALT) is an enzyme found mainly in the cytosol of hepatocytes, with much higher levels of ALT activity demonstrable in the liver than in other tissues [1–3]. Non-alcoholic fatty liver disease (NAFLD) including non-alcoholic steatohepatitis (NASH) is the most common cause of abnormally high levels of ALT [4–8]. NAFLD and NASH are highly prevalent amongst patients with obesity [9–12] and approximately 50% of people affected by obesity have elevated levels of ALT and co-existing NAFLD [13]. Elevated ALT is an independent predictor of prevalent NAFLD and there is a significant association between elevated ALT and NASH and liver fibrosis [14]. Most interventions to reduce body weight in patients who are overweight or obese tend to lead to improvements in NAFLD. For example, after bariatric surgery improvements in NASH and reductions in ALT have been described after 12 months [15]. Conversely, patients with severe obesity undergoing omega loop gastric bypass had a significant increase in ALT whereas those undergoing sleeve gastrectomy or Roux-en-Y gastric bypass had a reduction in ALT [16]. More severe deteriorations in NAFLD after bypass bariatric surgery have also been described, but are the exception rather than the rule [17]. Weight loss interventions based on either increased physical activity, dietary restriction or a combination of the two, have been shown to reduce ALT in meta-analyses [18, 19]. However, intensive caloric restriction such as with a very low-calorie diet has been associated with increased ALT in one study [20].

The underlying mechanistic basis for the heterogeneity in the hepatic physiological response to weight loss interventions has not previously been determined. Nor have previous studies examined serial changes in ALT over time in bariatric patients after initiation of a dietary weight loss intervention. While no substitute for quantifying steatosis with radiological imaging or spectroscopy, changes in liver enzymes could yield valuable insights into the mechanistic basis for improvements in metabolic health with dietary restriction. We have previously described changes in body weight and metabolic characteristics in adults with severe obesity undergoing a milk-based meal replacement programme in our centre [21]. We used milk as the basis for our meal replacement programme for several reasons. It is a relatively low-cost alternative to commercially produced meal replacement supplements, which can be prohibitively cost ineffective [22]. Whey proteins in milk have been shown to attenuate muscle loss [23] and to preserve muscle fibre protein synthesis [24] during very low calorie diets. In men with obesity, milk has been shown to reduce appetite, dietary intake and body weight [25], as well as improving glucose and fat metabolism [26]. A recent trial showed that drinking low fat milk made children feel fuller and eat less later in the day compared to juice or water [27]. We sought to determine the influence of a 24-week milk-based meal replacement programme in patients with severe and complicated obesity on serum ALT as a putative marker of NAFLD.

## Methods

### Study design, population and setting

This was a single-center, retrospective cohort study, conducted in accordance with Strengthening The Reporting of Observational Studies in Epidemiology (STROBE) guidelines [28]. The study population included bariatric patients who were referred to our milk-based meal replacement programme. During the programme, patients attended the bariatric clinic every 2 weeks for 24 weeks (14 visits in total), met the nurse, dietitian and physician at each visit, had blood tests performed and had weight, height and blood pressure measurements taken. All baseline and follow-up measures for the programme were conducted in the Bariatric Medicine Clinic at the Centre for Diabetes, Endocrinology and Metabolism in Galway University Hospitals (GUH).

### Inclusion and exclusion criteria

Male and female patients aged 18 years or older, referred to the bariatric service for assessment of severe obesity were eligible for inclusion. Our clinical practice is to define severe obesity as a body mass index (BMI) ≥ 40 kg m^−2^ (or ≥ 35 kg m^−2^ with co-morbidities such as type 2 diabetes or obstructive sleep apnea syndrome). Patients must have been willing to attend all the 14 scheduled intervention visits. Females of childbearing potential who were pregnant, breast-feeding or intended to become pregnant or were not using adequate contraceptive methods were not considered eligible for the programme. Those with a recent myocardial infarction (within 6 months), untreated arrhythmia, untreated left ventricular failure, recent cholelithiasis (within the past year), hepatic or renal dysfunction, type 1 diabetes, untreated major psychiatric disorders, eating disorders, cancer, previous bariatric surgery, a BMI < 35 kg m^−2^ or those deemed unlikely to attend for the full programme (e.g. frequent clinic non-attendance) were excluded from the programme. Excess alcohol consumption, defined as more than 21 units per week for men and more than 14 units per week for women, was also an exclusion criterion. Moreover, patients were requested to avoid any alcohol whatsoever during the 6-month intervention. Patients with a history of lactose intolerance or allergy to milk or other dairy products were excluded from participation in the milk diet.

### Ethics approval

The study was approved by the Galway University Hospitals’ Central Research Ethics Committee in December 2017 (ref CA 1900). As the programme was part of standard clinical care for patients attending our service between 2013 and 2016 and was not a prospective research study, we did not prospectively obtain written informed consent from patients to use their data for research purposes. Considering recent changes in European legislation regarding the use of personal data [the General Data Protection Regulation (GDPR)[, we have only used data in this study from the subgroup of patients who agreed to this retrospectively and provided written informed consent.

### Intervention

The milk-based low energy liquid diet (LELD) consisted of three continuous 8-week phases, each with fortnightly visits to the bariatric medicine clinic, as previously described [21]. During the first (weight loss) phase from weeks one to eight inclusive, an exclusively milk-based liquid diet was prescribed, consisting of approximately 2.5 L per day of semi-skimmed milk (depending on calculated protein requirements [29, 30]) divided in seven portions throughout the day in equal doses, with additional sodium replacement, vitamin, mineral and fiber supplementation, equating to approximately 1200 kcal/day. The dietary composition of 100 mL of semi-skimmed milk included protein (3.5 g), carbohydrate (5 g, of which sugars 5 g) and fat (1.5 g). Throughout this phase, renal and liver profiles were assessed every 2 weeks and the patients were seen by a physician, bariatric nurse and dietitian at each visit. During the second phase (weight stabilization) from weeks nine to sixteen inclusive, there was a gradual re-introduction of low-calorie meals from a set menu over 8 weeks, according to protocol under the supervision of the bariatric dietitian with fortnightly visits continuing. During the third phase (weight maintenance) from weeks 17 to 24 inclusive, the milk component of the diet was stopped completely and a standard isocaloric diet was restarted, based on individualized meal plans, under the supervision of the bariatric dietitian.

### Measurements

Weight was measured on a Tanita® scale and height with a Seca® wall-mounted stadiometer, according to departmental standard operating procedures. After an overnight fast, bloods were drawn for glucose, liver, renal and lipid profiles. All blood samples were processed locally in the Galway University Hospitals’ Department of Clinical Biochemistry (certified to ISO 15189 2007 accreditation standard). ALT was quantitated using the Roche Cobas® 8000 enzymatic assay with spectrophotometric detection. The decrease in absorbance at 340 nm is directly proportional to the concentration of ALT. Inter-assay precision at a mean ALT concentration of 28 iu/L, 121 iu/L and 210 iu/L was 5.4%, 1.6% and 2%, respectively. All of the above measures were preformed fortnightly throughout the programme. Normal ALT reference ranges were taken from the American College of Gastroenterology (ACG) clinical guidelines for the evaluation of abnormal liver chemistries (29 to 33 iu/L in men, and 19 to 25 iu/L in women) [31].

SPSS version 25 was used for all statistical analyses. Summary data were presented as means and standard deviations for normally distributed data and medians and interquartile ranges for skewed data, while categorical variables were presented as numbers and percentages.

In addition to analyzing ALT levels across the entire cohort we also classified the patient population into two groups according to baseline ALT levels as ‘normal’ (patients with normal baseline ALT levels), and ‘high’ (patients with elevated baseline ALT levels). For baseline characteristics, the independent samples *t* test, was used to compare normally distributed variables between groups, while non-normally distributed variables were compared using the Mann–Whitney *U* test. Pearson’s chi square was used to compare proportions of categorical variables.

The Wilcoxon signed-rank test was used to compare changes in ALT levels from baseline to week 2, and from baseline to week 24, respectively. Changes in ALT overtime in weeks 0, 2, 4, 6, 8, 16, and 24 were analyzed using the Friedman test. Changes over time in weight, BMI, excess body weight percentage (EBW%), HbA1c, total, LDL and HDL cholesterol and triglycerides were analysed using repeated measures ANOVA.

## Results

### Baseline characteristics of study population

As described in our previous study [32], 260 patients were enrolled between January 2013 and Oct 2018 into the milk-based meal replacement programme at the Bariatric Medicine clinic in Galway University Hospitals. Of these, 139 (53.5%) completed all 24 weeks of the intervention, with 121 (46.5%) discontinuing the intervention. From 139 completers, 105 (75.5%) agreed to participate in this study and provided written informed consent. Given that 1867 new patients were seen in our bariatric service over the 6-year study period, 13.9% of newly referred bariatric patients ultimately participated in our milk programme.

Details of patients’ characteristics are shown in Table 1. The average age of participants was 51.1 ± 11.2 (range 18.0–71.6) years. 56 (53.3%) were female. The overall median baseline ALT level was 28.0 [20.0, 40.5] iu/L. The 48 patients with a high ALT had no statistically significant differences to the 57 with normal ALT in age, sex, anthropometric or metabolic variables.

**Table 1:** Baseline characteristics of patients completing the milk programme

Parameters	All patients	Normal ALT	High ALT	*P*
N (%)	105 (100%)	57 (54.3%)	48 (45.7%)	
Female	56 (53.3%)	33 (57.9%)	23 (47.9%)	0.307
Age (years)	51.1 ± 11.2	52.9 ± 9.4	49.1 ± 12.8	0.079
Height (m)	1.7 ± 0.1	1.7 ± 0.1	1.7 ± 0.1	0.502
Weight (kg)	144.0 ± 27.6	145.2 ± 27.3	142.6 ± 28.2	0.627
BMI (kg m^2^)	50.6 ± 8.0	51.5 ± 8.2	49.7 ± 8.0	0.254
EBW%	102.5 ± 32.0	106.0 ± 33.0	98.6 ± 31.2	0.254
ALT^a^ (iu/L)	28.0 [20.0,40.5]	22.0 [17.0,24.5]	41.5 [34.3,63.5]	< 0.0005
Total cholesterol (mmol/L)	4.6 ± 1.0	4.6 ± 1.0	4.6 ± 1.0	0.786
LDL (mmol/L)	2.5 ± 0.9	2.5 ± 0.9	2.6 ± 0.8	0.442
HDL (mmol/L)	1.2 ± 0.4	1.3 ± 0.4	1.2 ± 0.4	0.131
Triglyceride (mmol/L)	1.8 ± 0.8	1.7 ± 0.6	1.9 ± 1.0	0.151
Diabetes	37 (35.2%)	19 (33.3%)	18 (37.5%)	0.656
HbA1c (mmol/mol)	48.2 ± 15.6	48.1 ± 16.3	48.4 ± 15.0	0.943
*Medications*				
Metformin	38 (36.2%)	22 (38.6%)	16 (33.3%)	0.576
Insulin	10 (9.5%)	7 (12.3%)	3 (6.3%)	0.294
SU	15 (14.3%)	10 (17.5%)	5 (10.4%)	0.298
DPP4	9 (8.6%)	6 (10.5%)	3 (6.3%)	0.436
SGLT2	6 (5.7%)	3 (5.3%)	3 (6.3%)	0.828
GLP1	18 (17.1%)	9 (15.8%)	9 (18.8%)	0.688
PPARG	1 (1%)	1 (1.8%)	–	0.356
ARBs	25 (23.8%)	12 (21.1%)	13 (27.1%)	0.470
ACEIs	30 (28.6%)	19 (33.3%)	11 (22.9%)	0.239
Beta blockers	31 (29.5%)	17 (29.8%)	14(29.2%)	0.914
Alpha blockers	6 (5.7%)	3 (5.3%)	3 (6.3%)	0.828
Diuretics	31 (29.5%)	15 (26.3%)	16 (33.3%)	0.432
Ezetemibe	4 (3.8%)	2 (3.4%)	2 (4.2%)	0.861

Values are presented as Mean ± SD

Comparisons between groups were made using Independent samples *t* test for normally distributed data, and Mann–Whitney *U* test for non-normally distributed data

Proportions with categorical variables were compared using Pearson’s Chi-square test

*BMI* Body Mass Index, *EBW%* Excess Body Weight Percentage, *ALT* Alanine Aminotransferase, *LDL* Low Density Lipoprotein, *HDL* High Density Lipoprotein, *HbA1c* Glycated haemoglobin, *SU* Sulfonylureas, *DDP4* Dipeptidyl peptidase-4 inhibitors, *SGLT2* Sodium-glucose co-transporter 2 inhibitors, *GLP1* Glucagon-like peptide 1, *PPARG* Peroxisome proliferator-activated receptor gamma, *ARBs* Angiotensin II receptor blockers, *ACEIs* Angiotensin-converting enzyme inhibitors

^a^Denotes variables that are not normally distributed, presented as median [IQR]

### Changes in ALT, anthropometrics, and metabolic variables over 24 weeks

**Fig. 1:**
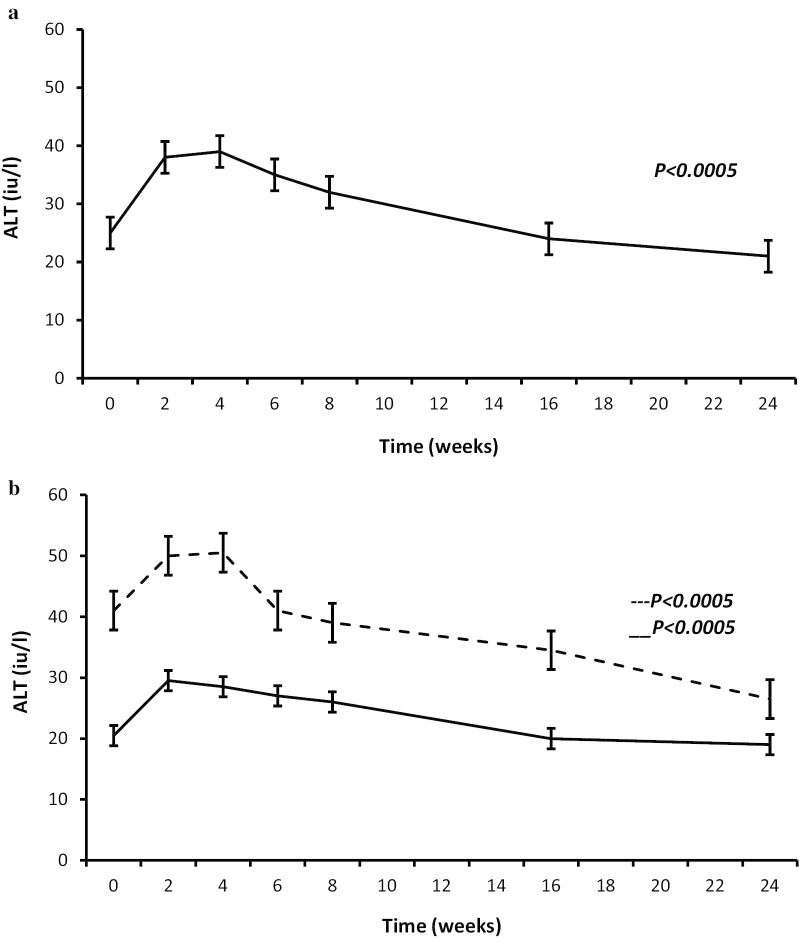
**a** Changes in ALT during milk-based meal replacement programme. Alanine aminotransferase (ALT). All values represent median and interquartile range. *P* significant for the trend. **b** Changes in ALT during milk-based meal replacement programme in patients with high versus normal baseline ALT. Dashed line represents high ALT group. Continuous line represents normal ALT group. All values represent median and interquartile range. The Friedman test was used to analyse changes in ALT over time. *P* significant for the trend

Table 2 describes the changes in anthropometric variables and ALT levels at weeks 2, 4, 6, 8, 16, and 24 of the programme. From baseline to week 2, ALT levels significantly increased from 28.0 [20.0, 40.5] to 40.0 [26.0, 55.0] iu/L (*p* < 0.0005), while from baseline to week 24, there was a statistically significant overall reduction in ALT from 28.0 [20.0, 40.5] iu/L to 21.0 [17.0, 28.3] iu/L (*p* < 0.0005). Over time ALT increased from a baseline value of 25 [20.0, 37.3] iu/L to 38 [26.0, 53.5], and 39 [26.0, 60.0] iu/L in weeks 2 and 4, respectively, followed by a significant subsequent reduction to 35 [26.0, 49.0], 32 [23.0, 41.3], 24 [19.0, 34.3], and 21 [17.0, 28.3] iu/L (*p* < 0.0005) at weeks 6, 8, 16, and 24, respectively (Table 2; Fig. 1a). When we compared patients with normal versus high baseline ALT levels we found that the rise in ALT was greater in those with high baseline ALT, in whom ALT rose from 41.5 [34.3, 63.5] iu/L to 51.0 [41.0,78.0] iu/L (*p* = 0.001) at week 2, then decreased to 27.0 [21.0, 33.5] iu/L (*p* < 0.0005) by 24 weeks. Taking a slightly different analytical approach and using the Friedman test (the non-parametric equivalent of analysis of variance), similar results were obtained with ALT increasing from 41.0 [33.0, 62.5] iu/L at baseline to 50.0 [40.5, 85.0], and 50.5 [39.5, 68.3] iu/L at weeks 2, and 4, respectively, reaching 27.0 [21.0, 33.5] iu/L (*p* < 0.0005) by 24 weeks. In contrast, those with normal baseline ALT levels had a non-significant increase in ALT from 20.5 [17.0, 24.0] iu/L to 29.5 [20.3, 41.0] iu/L at week 2, followed by a subsequent reduction to 19.0 [16.0, 22.0] iu/L, (*p* < 0.0005) by 24 weeks (Table 2; Fig. 1b). As described in this cohort previously [32], there were significant reductions in HbA1c in patients with and without type 2 diabetes (Table 2).

**Table 2:** Changes in anthropometric and metabolic variables over time in patients with normal and high baseline ALT completing the milk programme

	Week 0	Week 2	Week 4	Week 6	Week 8	Week 16	Week 24	*p*
ALT (iu/L)^a^	25 [20.0,37.3]	38 [26.0,53.5]	39 [26.0,60.0]	35 [26.0,49.0]	32 [23.0,41.3]	24 [19.0,34.3]	21 [17.0,28.3]	< 0.0005
ALT (iu/L)^ab^	41.0 [33.0,62.5]	50.0 [40.5,85.0]	50.5 [39.5,68.3]	41.0 [34.0,67.3]	39.0 [32.0,51.0]	34.5 [25.0,41.3]	26.5 [21.0,36.5]	< 0.0005
ALT normal (iu/L)^ac^	20.5 [17.0,24.0]	29.5 [20.3,41.0]	28.5 [20.3,43.0]	27.0 [21.3,38.5]	26.0 [18.3,36.0]	20.0 [17.0,25.0]	19.0 [16.0,22.0]	< 0.0005
Weight (kg)	144.2 ± 28.0	137.6 ± 27.4	134.1 ± 27.0	131.0 ± 26.2	128.3 ± 25.6	123.0 ± 25.0	121.6 ± 25.4	< 0.0005
BMI (kg m^2^)	50.7 ± 8.1	47.4 ± 10.4	45.4 ± 12.0	44.5 ± 12.0	45.2 ± 7.6	43.2 ± 7.5	43.0 ± 7.6	< 0.0005
EBW (%)	103.1 ± 32.4	89.6 ± 42.0	81.6 ± 48.2	78.3 ± 47.4	81.0 ± 30.6	73.0 ± 30.0	71.0 ± 31.0	< 0.0005
HbA1c (mmol/mol)^d^	39.3 ± 4.0	37.6 ± 4.0	37.0 ± 4.0	36.3 ± 4.0	36.0 ± 4.2	35.4 ± 3.3	35.3 ± 3.5	< 0.0005
HbA1c (mmol/mol)^e^	67.2 ± 13.3	62.2 ± 13.0	59.0 ± 13.0	56.0 ± 14.2	54.0 ± 15.0	48.5 ± 14.4	48.6 ± 14.1	< 0.0005
LDL (mmol/L)	2.6 ± 1.0	2.2 ± 1.0	2.1 ± 0.7	2.2 ± 1.0	2.2 ± 1.0	2.5 ± 1.0	2.6 ± 1.0	< 0.0005
HDL (mmol/L)	1.2 ± 0.4	1.1 ± 0.3	1.1 ± 0.3	1.1 ± 0.3	1.1 ± 0.3	1.2 ± 0.3	1.3 ± 0.4	< 0.0005
Triglyceride (mmol/L)	1.8 ± 0.7	1.5 ± 0.6	1.4 ± 0.5	1.4 ± 0.5	1.3 ± 0.5	1.3 ± 0.5	1.3 ± 0.5	< 0.0005
Total cholesterol (mmol/L)	4.6 ± 1.0	3.9 ± 1.0	3.7 ± 1.0	3.8 ± 1.0	4.0 ± 1.0	4.2 ± 1.0	4.4 ± 1.0	< 0.0005

Values are presented as Mean ± SD

^a^Denotes variables that are not normally distributed presented as median [IQR]

^b^Denotes variables that represent values higher than normal at the start of the programme

^c^Denotes variables that represent values that were normal at the start of the programme

^d^Denote patients without history of type 2 diabetes

^e^Denote patients with history of type 2 diabetes

## Discussion

In a cohort of patients with severe and complicated obesity completing a milk-based meal replacement programme with substantial weight loss over 24 weeks, we found a significant, early and unanticipated rise in ALT that was particularly pronounced in patients who had an elevated ALT at the start of the intervention. This was followed by an overall net reduction in ALT after 24 weeks. To the best of our knowledge this is the first description of a “monophasic” ALT response over multiple time-points with dietary restriction in patients with severe and complicated obesity.

Our findings suggest potential worsening of hepatic inflammation and ectopic fat deposition early in the course of dietary weight loss interventions, prior to longer-term improvements in liver health. However it must be emphasised that this assertion is speculative, because ALT is regarded as a “modestly good at best” indicator of hepatocyte damage in patients with NAFLD [13]. There have been contradictory findings from previous studies on the response of ALT to different types of weight-loss interventions [15–20]. Our findings also suggest that patients with elevated ALT (suggesting a degree of NAFLD) are at higher risk of additional hepatic inflammation, at least during the early stages of dietary restriction-based weight loss interventions.

A recent study of patients undergoing a very low-calorie diet (VLCD) for 3 weeks prior to bariatric surgery noted a transient rise in ALT [20]. This and other studies [16] have the limitation of measuring ALT levels at two time-points only, whereas we have described short and medium term changes at several time-points, during an acute weight loss phase and then a subsequent period of less intensive weight loss. This approach has allowed us to demonstrate both the acute rise in ALT associated with the early weight loss phase of our LELD intervention as well as the subsequent reduction in ALT levels in the aftermath of significant weight loss once weight had started to stabilise. Ultimately, our findings help to explain the apparently conflicting data on ALT levels and weight loss interventions in the literature to date, suggesting that ALT rises during acute and significant weight loss but that once this phase is over and weight stabilises then ALT levels typically fall below baseline levels. This is consistent with previous findings in a post-hoc analysis of a Danish very low calorie diet intervention (VLCD, ~ 800 kcal/day) [33] which noted a transient rise in liver transaminases in women but not in men.

The exact mechanisms driving the rise in ALT levels during the early weeks of weight loss interventions are unclear and are beyond the scope of this study. It has previously been demonstrated that during fasting, increased adipose-derived non-esterified fatty acid (NEFA) flux to the liver occurs, promoting intra-hepatic fat accumulation [34–36]. Increased intrahepatic fat, in turn, has been associated with increased generation of fatty acids within the liver via de novo lipogenesis [37]. In a similar manner, studies of male livers following 48 h of fasting have reported evidence of significant triglyceride accumulation [38]. It may be that states of reduced caloric intake such as those achieved during the weight loss phase of our intervention or after bariatric surgery are associated with an increase in intrahepatic fat concentrations, which in turn may drive increases in ALT levels and potentially NAFLD severity. Once caloric intake and weight has stabilised, this effect may then decrease and intra-hepatic fat accumulation may diminish, leading to an eventual net decrease in ALT levels from baseline.

Aside from fat mobilisation and lipogenesis, another potential mechanism that could lead to increased ALT during the milk diet is the activation of hepatic gluconeogenesis during caloric restriction, as has been demonstrated in mouse studies [39]. Bearing in mind that liver disease was an exclusion criterion for the intervention and that patients were required not to consume any alcohol throughout the 6 months, we think that these are unlikely factors to account for the transient rise in ALT. Moreover, incident drug use such as with non-steroidal anti-inflammatory drugs or antibiotics or very strenuous physical activity, while not formally quantified in this study, are unlikely to account for our findings.

The reasons for the relatively larger rise in ALT in patients who had elevated levels at baseline are unclear but may reflect an inherent vulnerability to accumulation of excess intrahepatic lipid at any given level of excess body fat accumulation. This heterogeneity in the relationship between adiposity and metabolic dysfunction is likely to arise from a complex interaction of genetic and environmental factors and needs to be explored further in mechanistic studies, incorporating quantification of insulin sensitivity, fat mass, changes in circulating non-esterified fatty acids and other relevant confounders.

This study has a number of limitations, not least its retrospective nature and its inclusion of patients who completed the milk diet programme, from which there was a very high attrition rate, with no data available on patients who dropped out. Thus, our findings are not generalisable to other patient populations outside of a hospital-based cohort of patients with severe and complicated obesity, nor are they generalisable to other weight loss interventions. Arguably our most important limitation is the use of ALT as a surrogate indicator of NAFLD, as it is relatively non-specific and lacks sensitivity [40]. Again, the use of imaging and histological studies would help to address this limitation in future studies. These were not acquired as part of the routine clinical assessment of our patients enrolling in the milk programme. Of note, previous studies have utilised ALT levels as a surrogate marker of NAFLD [13, 41, 42]. Moreover, we think that our inclusion and exclusion criteria for the milk-based meal replacement programme would have reduced the risk of confounding (for example from excess alcohol consumption), making it more likely that variations in ALT levels within our population reflected changes in hepatic fat accumulation and inflammation. One other limitation is the 6-month duration of the study. Future studies could focus on longer-term follow-up, in particular with regards to changes in ALT levels associated with weight regain.

## Conclusion

In adults with severe and complicated obesity undergoing a milk-based meal replacement programme, we demonstrated an initial significant rise in ALT levels over the first 8 weeks which was greater in those with elevated ALT levels at baseline. This was followed by an overall reduction in ALT levels during the weight stabilisation and maintenance phases of our programme such that there was a net overall decrease in ALT levels at 24 weeks. Whether these preliminary and exploratory observations are related to changes in liver fat content needs to be determined by imaging studies, but this seems biologically and mechanistically plausible. These results may be of particular relevance to patients with severe hepatic inflammation undergoing acute dietary restriction for weight loss, where vigilance relating to the use of hepatotoxic drugs could be particularly important. The mechanistic factors underlying the heterogeneity in the response of intrahepatic lipid to dietary weight loss warrant further scientific scrutiny.

## Data Availability

The data can be made available upon request.

## Abbreviations

ACEIs: Angiotensin-converting enzyme inhibitor
ACG: American College of Gastroenterology
ARBs: Angiotensin II receptor blockers
ALT: Alanine aminotransferase
ANOVA: Analysis of variance
BMI: Body mass index
DDP4: Dipeptidyl peptidase-4 inhibitors
EBW%: Excess body weight percentage
GDPR: The General Data Protection Regulation
GLP1: Glucagon-like peptide 1
GUH: Galway University Hospitals
HbA1c: Glycated haemoglobin
HDL: High Density Lipoprotein
IQR: Interquartile range
LELD: The milk-based low energy liquid diet
LDL: Low Density Lipoprotein
NAFLD: Non-alcoholic fatty liver disease
NASH: Non-alcoholic steatohepatitis
NEFA: Adipose-derived non-esterified fatty acid
PPARG: Peroxisome proliferator-activated receptor gamma
SGLT2: Sodium-glucose co-transporter 2 inhibitors
STROBE: Strengthening the Reporting of Observational Studies in Epidemiology
SU: Sulfonylureas
VLCD: Very low calorie diet
